# Plasma Triglyceride Levels May Be Modulated by Gene Expression of *IQCJ*, *NXPH1*, *PHF17* and *MYB* in Humans

**DOI:** 10.3390/ijms18020257

**Published:** 2017-01-26

**Authors:** Bastien Vallée Marcotte, Frédéric Guénard, Hubert Cormier, Simone Lemieux, Patrick Couture, Iwona Rudkowska, Marie-Claude Vohl

**Affiliations:** 1 Institute of Nutrition and Functional Foods (INAF), Laval University, 2440 Hochelaga Blvd, Quebec, QC G1V 0A6, Canada; bastien.vallee-marcotte.1@ulaval.ca (B.V.M.); frederic.guenard@fsaa.ulaval.ca (F.G.); hubert.cormier.1@ulaval.ca (H.C.); simone.lemieux@fsaa.ulaval.ca (S.L.); patrick.couture@crchudequebec.ulaval.ca (P.C.); 2 CHU de Québec Research Center–Endocrinology and Nephrology, 2705 Laurier Blvd, Quebec, QC G1V 4G2, Canada; iwona.rudkowska@crchudequebec.ulaval.ca

**Keywords:** gene-diet interactions, plasma lipid levels, ω-3 fatty acids, genome-wide association study, nutrigenetics, epigenetics

## Abstract

A genome-wide association study (GWAS) by our group identified loci associated with the plasma triglyceride (TG) response to ω-3 fatty acid (FA) supplementation in *IQCJ*, *NXPH1*, *PHF17* and *MYB*. Our aim is to investigate potential mechanisms underlying the associations between single nucleotide polymorphisms (SNPs) in the four genes and TG levels following ω-3 FA supplementation. 208 subjects received 3 g/day of ω-3 FA (1.9–2.2 g of EPA and 1.1 g of docosahexaenoic acid (DHA)) for six weeks. Plasma TG were measured before and after the intervention. 67 SNPs were selected to increase the density of markers near GWAS hits. Genome-wide expression and methylation analyses were conducted on respectively 30 and 35 participants’ blood sample together with in silico analyses. Two SNPs of *IQCJ* showed different affinities to splice sites depending on alleles. Expression levels were influenced by genotype for one SNP in *NXPH1* and one in *MYB*. Associations between 12 tagged SNPs of *IQCJ*, 26 of *NXPH1*, seven of *PHF17* and four of *MYB* and gene-specific CpG site methylation levels were found. The response of plasma TG to ω-3 FA supplementation may be modulated by the effect of DNA methylation on expression levels of genes revealed by GWAS.

## 1. Introduction

Cardiovascular diseases (CVD) are known to have several causes, including environmental and genetic predispositions. Nutrition is an important environmental factor affecting the risk of developing CVD, and several dietary recommendations have been issued for prevention. However, these recommendations currently do not take into account the fact that individuals respond differently to dietary interventions although this phenomenon is well documented in the literature [1]. For instance, it is recognized that ω-3 fatty acids (FA) from marine sources, including eicosapentaenoic acid (EPA) and docosahexaenoic acid (DHA), exert beneficial effects on cardiovascular health owing, among others, to their hypotriglyceridemic properties [2]. However, a large inter-individual variability of the metabolic response to an ω-3 FA supplementation has been observed. In the European FINGEN study, 31% of participants who received a supplement of 1.8 g/day of ω-3 FA for eight weeks did not reduce their plasma triglyceride (TG) levels [3]. Similarly, our research group reported that 29% of all participants of the Fatty Acid Sensor (FAS) Study, who received ω-3 FA supplementation providing 5 g of fish oil per day (1.9–2.2 g of EPA and 1.1 g DHA) over a period of six weeks did not have their plasma TG levels decreased [4,5]. This heterogeneity of the plasma TG response is partly due to genetic factors, including gene-diet interactions [1,6].

Research groups around the world have been studying genes associated with lipid metabolism such as apolipoprotein E and peroxisome proliferator-activated receptor α, and discovered several single nucleotide polymorphisms (SNPs) implicated in this phenomenon [3,7,8,9,10]. However, these variations explain a very small proportion of the variance of the plasma TG levels in response to ω-3 FA supplementation. Since many other associated variations may remain unknown, our team recently conducted a genome-wide association study (GWAS) on subjects of the FAS Study and identified 13 SNPs associated with the plasma TG response [6]. A genetic risk score derived with these SNPs explained 21.5% of the variance of the plasma TG response [6]. Due to linkage disequilibrium (LD) between SNPs in neighbouring genes, it is difficult to discern actual causative SNPs solely with GWAS results. We therefore recently increased the density of markers at TG response-associated loci for the IQ motif-containing J (*IQCJ*), neurexophilin-1 (*NXPH1*), PHD finger protein 17 (*PHF17*) and V-Myb avian myeloblastosis viral oncogene homolog (*MYB*) genes, and found numerous gene-diet interactions and effects of SNPs in the *IQCJ*, *NXPH1* and *MYB* genes on plasma TG levels [11]. However, the underlying mechanisms relating these genes and lipid metabolism remain unclear. *IQCJ* and *NXPH1* are known for their functions in the central and peripheral nervous systems [12,13,14,15]. *PHF17* is involved in mitosis, meiosis and tumor suppression [16,17]. *MYB* regulates various cell functions and plays an important role in hematopoiesis and cancer development [18,19]. Yet, very little associations between these genes and lipid metabolism have been established. Thus, the purpose of this study is to investigate the mechanisms underlying associations between SNPs of candidate genes and TG levels following ω-3 FA supplementation by analyzing gene expression, DNA methylation levels and conducting in silico analyses.

## 2. Results

Table 1 shows the baseline characteristics of the participants. In accordance with the inclusion criteria, the average body mass index (BMI) pre- and post-supplementation of all subjects was >25 kg/m^2^. The average pre-supplementation plasma TG level was above the cut-point value of 1.129 mmol/L based on the last recommendations of the American Heart Association [2].

Table 2 shows allele frequencies of tagged SNPs. All SNPs were in Hardy-Weinberg equilibrium (HWE). 87% of the genetic variability of *IQCJ*, as well as 85% of *NXPH1*, 96% of *PHF17* and 100% of *MYB* were covered. Most of the SNPs were located in introns, except for three SNPs of *PHF17* that were located in its upstream region, another of *PHF17* that was in the three prime untranslated region (3′ UTR) and one of *MYB* that was in its downstream region.

We searched for possible connections between *IQCJ*, *NXPH1*, *PHF17* and *MYB* and lipid metabolism by conducting in silico analyses. First, RNA splicing analyses revealed two SNPs of *IQCJ*, rs2595260 and rs9827242, showing different affinities to splice sites depending on alleles. Besides, LD analyses indicated no LD between tagged SNPs and other SNPs located in coding or promoter regions. Transcription factor affinity predictions demonstrated the poor affinity of tagged SNPs with transcription factors. We also investigated expression levels of *IQCJ*, *NXPH1*, *PHF17* and *MYB* in specific tissues using the Tissue-specific Gene Expression and Regulation (TiGER) database and the Expression Atlas. *IQCJ* was especially expressed in the brain. Similarly, *NXPH1* was expressed in the brain, but also in the peripheral nervous system. *PHF17* was more ubiquitous. It was mostly expressed in the small intestine, prostate, mammary gland and kidney. *MYB* was expressed in bone marrow, thymus, testis and blood. Analyses of the expression microarrays revealed several effects of genotype on expression levels (Table 3). Two SNPs, namely rs10486228 (*NXPH1*) and rs17639758 (*MYB*), were significantly associated with gene expression levels. Three other SNPs, rs17782879 in *IQCJ*, rs11769942 in *NXPH1* and rs11154794 in *MYB*, had marginal but non-significant effects on gene expression. No associations were observed between SNPs in *PHF17* and corresponding gene expression levels.

Significant associations of tagged SNPs from GWAS-associated genes with methylation levels are presented in Table 4. A total of 17 significant associations were found with CpG sites and *IQCJ* SNPs. There were also 71 significant associations with *NXPH1* SNPs, 15 with *PHF17* SNPs and seven with *MYB* SNPs. Because of the large number of results and the number of statistical tests computed, we accounted for multiple testing with a false discovery rate. 6 SNPs; rs2044704, rs1962071, rs2595260, rs1449009, rs2621309 and rs61332355, all in *IQCJ*, remained significant after false discovery rate correction.

Spearman correlation coefficients between gene expression and CpG site methylation levels are presented in Table 5. Significant correlations between gene expression and methylation levels were obtained with two CpG sites of *IQCJ* (cg15736726 and cg23982461), one of *NXPH1* (cg06328127), three of *PHF17* (cg04482257, cg27628849 and cg17233452) and two of *MYB* (cg01369646 and cg02127509).

Spearman correlation coefficients between TG levels and CpG site methylation levels are presented in Table 6. Significant correlations between gene expression and methylation levels were obtained with one CpG site of *IQCJ* (cg09784347), three of *NXPH1* (cg00852549, cg00399951 and cg20378002), four of *PHF17* (cg04482257, cg09863040, cg12832492 and cg01223512) and two of *MYB* (cg18253802 and cg07363239). As to correlations between TG levels and expression levels, the NM_001042705.1 transcript in *IQCJ* was significantly correlated with pre-supplementation TG levels (*p* = 0.0163).

## 3. Discussion

Our research group recently revealed, in a GWAS, potential SNPs associated with the plasma TG response to ω-3 FA supplementation [6]. We increased the density of markers around GWAS-associated SNPs in the *IQCJ*, *NXPH1*, *PHF17* and *MYB* genes to further verify whether they are associated with plasma TG levels following ω-3 FA supplementation [11]. We found several effects of genotype and gene-diet interactions on plasma TG levels [11]. In the present study, we analysed gene expression and methylation levels, and conducted in silico analyses in order to better understand the potential underlying mechanisms by which *IQCJ*, *NXPH1*, *PHF17* and *MYB* interact with plasma ω-3 FA supplementation to modulate plasma TG levels.

We first observed that two SNPs of *IQCJ*, rs2595260 and rs9827242, had different affinities with splice sites depending on alleles. In our previous study, these SNPs were also found to exert significant gene-diet interactions modulating plasma TG levels [11]. These results therefore indicate that an individual’s genotype may possibly affect gene expression of *IQCJ* through the alteration of RNA splicing. Likewise, there was a marginal association between one SNP of *IQCJ*, rs17782879, and its expression levels. We did not find any other substantial results in RNA splicing analyses, nor did we find specific affinities of tagged SNPs with transcription factors. This can be attributable to the location of tagged SNPs. Accordingly, the vast majority of these SNPs are located within introns, whereas regulatory regions are located in splice sites, promoter regions, 3′ UTR, near gene regions (3′ or 5′), and transcribed DNA sequences are located in exons. We therefore searched for other relevant SNPs in LD with tagged SNPs. However, none of these tagged SNPs were in LD with any other SNPs located in coding or promoter regions that have previously been associated with lipid metabolism or with SNPs previously identified in a lipid traits-related GWAS.

Additionally, according to in silico analyses, all candidate genes were poorly expressed in tissues related to lipid metabolism, such as adipose tissue, liver or pancreas. These observations are coherent with known functions of these genes. In fact, very few links between *IQCJ*, *NXPH1*, *PHF17*, *MYB* and lipid metabolism have been made in the past. *IQCJ* is bound to the schwannomin-interacting protein 1 (*SCHIP1*) to form another transcriptional unit (*IQCJ-SCHIP1*) [12]. *IQCJ-SCHIP1* plays roles in the function of initial axon segments and nodes of Ranvier, which are essential structures to saltatory conduction in the central and peripheral nervous systems [13]. According to the results of a GWAS conducted on participants of the Genetics of Lipid Lowering Drugs and Diet Network (*GOLDN*) study, which aimed to study the effect of lipid-lowering therapy to fenofibrate on the response of various lipid traits, *IQCJ-SCHIP1* may actually be associated with TG levels as well as very-low-density lipoprotein (VLDL) particle clearance [20]. VLDL particle clearance is an important determinant of blood TG levels [21]. Dysfunctional VLDL hydrolysis can increase TG-rich lipoprotein remnant particles synthesis, which is associated with an increased risk of atherosclerosis [21,22,23]. In the same GWAS, several SNPs of *NXPH1* were associated with type 2 diabetes, blood pressure and plasma LDL-cholesterol, HDL-cholesterol, TG as well as C-reactive protein levels [20]. In another study, *NXPH1* was associated with type 2 diabetes traits among a population of obese Hispanic children [24]. *NXPH1* is known to code for a neuronal glycoprotein that binds to α-neurexin to regulate neuronal function, neurotransmission, signalisation and various cell interactions [14,15,25]. *PHF17* codes for the Jade-1 protein. This protein interacts with histone acetyltransferase HBO1 and promotes histone acetylation in chromatin to regulate DNA replication and gene transcription [16,26,27]. *MYB* encodes the c-Myb transcription factor, which regulates the expression of a wide variety of genes involved in cell function [18]. *MYB* has been reported to be associated with the development of CVD, intracellular accumulation of lipid, differentiation of mesenchymal stem cells from adipose tissue, intestinal absorption of nutrients and possibly BMI [28,29,30,31]. The c-Myb protein is expressed in the vascular smooth muscle and it is induced by homocysteine, while high levels of homocysteine are recognized as an independent CVD risk factor [28,32].

Moreover, we tested whether tagged SNPs are associated with pre-supplementation methylation levels and observed several significant associations. Many tagged SNPs that were marginally or significantly associated with plasma TG levels and the plasma TG response to ω-3 FA supplementation in our previous study appear now to be significantly associated with methylation levels as well. This is particularly true for *IQCJ* and *NXPH1*. Mostly all SNPs of *IQCJ* showing significant gene-diet interactions are associated with one particular mutual CpG site (cg16975599). This CpG site is close to rs548649590, which was not tested for associations with plasma TG levels in our previous study. This SNP is not in LD with any SNP previously associated with plasma TG levels either. Furthermore, we accounted for multiple testing with a false discovery rate and found out that the strongest associations were in *IQCJ*. None of the SNP of *MYB* that was previously found to exert effects of gene-diet interactions on plasma TG levels was associated with methylation levels at CpG sites. None was in LD with any SNPs associated with CpG sites either. These outcomes suggest that the modulation of plasma TG levels through genes revealed by GWAS, especially *IQCJ* and *NXPH1*, may be regulated by DNA methylation, which is here influenced by genotype. As DNA methylation is strongly related to gene expression, individuals’ genotype could directly upregulate or downregulate gene expression [33,34]. Gene expression analyses also support this rationale. Indeed, gene expression of *IQCJ*, *NXPH1* and *MYB* appeared to be affected by DNA variations within these genes. It was also shown to be influenced by TG levels and methylation levels in *IQCJ*, *NXPH1*, *PHF17* and *MYB*.

## 4. Materials and Methods

### 4.1. Study Population

A total of 254 participants were recruited to participate in the FAS Study between September 2009 and December 2011 in the Quebec City metropolitan area through advertisements in local newspapers and electronic messages sent to university students and employees. Participants had to be between 18 and 50 years of age, to be non-smokers and to have a BMI between 25 and 40 kg/m^2^. Candidates were excluded if they had taken fish oil supplements for at least six months prior to the beginning of the study or if they were suffering from any thyroid or metabolic disorders such as diabetes, hypertension, dyslipidemia or coronary heart disease. Statistical analyses were conducted on 210 subjects who completed the supplementation period. Data on plasma TG levels were missing for two of them, leaving 208 individuals in the final study sample.

### 4.2. Study Design and Diets

The complete study design and diets have been reported in previous papers [6,11]. Briefly, participants followed a run-in period of two weeks, wherein a trained registered dietitian gave dietary instructions for them to achieve the recommendations from Canada’s Food Guide, to ensure constant dietary intake and to keep a stable body weight throughout the protocol. Subsequently, they received ω-3 FA capsules (Ocean Nutrition, Dartmouth, NS, Canada) in a sufficient amount to cover the six-week supplementation protocol. Each capsule contained 1 g of fish oil concentrate. They had to take five capsules a day, providing 3 g of ω-3 FA, including 1.9–2.2 g of EPA and 1.1 g of DHA. They had to report any deviation from the protocol as well as experienced side effects. They also had to note their alcohol and fish consumption if any. Finally, oral and written dietary instructions were given to participants before each phase.

### 4.3. Anthropometric Measurements

Height and body weight were measured before the run-in period, as well as before and after the intervention following the recommendations of the Airlie Conference. BMI (kg/m^2^) was obtained by dividing weight (kg) by the squared height (m^2^).

### 4.4. Laboratory Methods

#### 4.4.1. Plasma Lipids

Methods to measure plasma lipids have previously been detailed [6,11]. Briefly, blood samples were collected after a 12 h overnight fast and 48 h alcohol abstinence. Blood samples were taken before the run-in period to verify whether individuals were presenting any metabolic disorders. Blood samples of remaining participants were taken before and after the ω-3 FA supplementation period. Enzymatic assays were used to measure plasma total cholesterol and TG concentrations [35,36].

#### 4.4.2. SNP Selection and Genotyping

SNPs were identified using the International HapMap Project SNP database, based on the National Center for Biotechnology information (NCBI) B36 assembly Data Rel 28, phase II + III, built 126. The Gene Tagger procedure in Haploview v4.2 was used to identify tagging SNPs with a minor allele frequency (MAF) >5% and pairwise tagging (*r*^2^ ≥ 0.80) located in gene regions and surrounding regions (2 kb upstream and downstream gene). The mean LD (*r*^2^) between SNPs was 0.96 for *IQCJ*, 0.96 for *NXPH1*, 0.97 for *PHF17* and 0.95 for *MYB*. SNPs were selected in a way to cover ≥85% of all common variations (MAF > 5%). The GenElute Gel Extraction Kit (Sigma-Aldrich Co., St. Louis, MO, USA) was used to extract genomic DNA (gDNA) from blood samples. Tagged SNPs were genotyped using TaqMan technology: 2.5 µL of each gDNA (40 ng/µL) and 2.5 µL of OpenArray Genotyper Master Mix (Life Technologies, Carlsbad, CA, USA) were mixed in a 384-well plate with validated primers and loaded onto genotyping plates using the QuantStudio^TM^ 12K Flex OpenArray^®^ AccuFill^TM^ System (Life Technologies). The QuantStudio^TM^ 12K Flex Real-Time PCR System (Life Technologies) was used for genotyping. Finally, TaqMan Genotyper v1.3 (Life Technologies) was used to call genotypes and export data.

#### 4.4.3. In Silico Analyses

RNA splicing analyses were performed for each tagged SNP using ESEfinder and Berkeley Drosophilia Genome Project splice site prediction tools [37,38,39]. LD between tagged and other non-tagged SNPs was assessed using SNP Annotation and Proxy Search (SNAP) with an *r*^2^ threshold of 0.8 and a distance limit of 500 kb [40]. LD was calculated with the Northern Europeans from Utah (CEU) population. We also used the Transcription factor Affinity Prediction (TRAP) web tools for single sequences to measure the possible transcription factor binding affinities to tagged SNP regions in the presence or absence of SNPs [41,42,43]. Furthermore, we used the TiGER database as well as the Expression Atlas to evaluate expression levels of our genes of interest in different tissues [44,45,46,47].

#### 4.4.4. Transcriptomic Gene Expression Analyses

The expression of transcripts associated with the four genes of interest was measured in peripheral blood mononuclear cells of the first 30 participants of the FAS study to complete the intervention via the Human-6 v3 Expression BeadChips (Illumina, San Diego, CA, USA). Transcriptomic analyses have previously been detailed [5].

#### 4.4.5. DNA Methylation Analyses

DNA methylation analyses were conducted on blood cells of 35 participants of the FAS study. Bisulfite conversion was made on 1 µg of DNA. Quantitative DNA methylation analyses were conducted at the Génome Québec Innovation Centre and McGill University (Montreal, QC, Canada). The Infinium HumanMethylation450 BeadChip (Illumina) was used for methylation sites coverage following the manufacturer’s instructions. CpG site positions were located according to genome build 37. The GenomeStudio software version 2011.1 (Illumina) as well as the methylation module were used to visualise and analyse methylation data. The ratio of signal intensity of the methylated alleles to the sum of methylated and unmethylated intensity signals of the alleles (β value = C/(T + C)) was used to estimate methylation levels (β value). Internal control probe pairs was applied for data correction. CpG sites were extracted using the GenomeStudio Methylation Module. A total of 10 CpG sites in *IQCJ*, 43 in *NXPH1*, 34 in *PHF17* and 24 in *MYB* were used.

#### 4.4.6. Statistical Analyses

The ALLELE procedure in SAS Genetics Statistical Software v9.3 (SAS Institute, Cary, NC, USA) was used to assess genotype distribution for any deviation from the HWE and to calculate the MAF. All other statistical analyses were performed in SAS v9.2. Normal distribution was evaluated with the box-plot, skewness and kurtosis ranges. Abnormally distributed variables were log_10_-transformed. Common genotype homozygotes, heterozygotes and rare genotype homozygotes were analysed separately in an additive model. However, rare genotype homozygotes showing a genotype frequency <5% were merged with heterozygotes for statistical analyses in a dominant model. The GLM procedure adjusted for age, sex and BMI was used to test for associations between pre-supplementation methylation levels and tagged SNPs for each gene independently. It was also used to test for the effects of genotype on gene expression levels using again age, sex and BMI as co-variates in the model. The MULTTEST procedure was then used to account for multiple testing controlling for false discovery rate. The CORR procedure adjusted for age, sex and BMI was used to correlate gene expression and methylation levels on 29 subjects using Spearman rank correlation. It was also used to correlate TG levels with gene expression and methylation levels on respectively 30 and 35 subjects using Spearman rank correlation. Statistical significance was fixed at *p* ≤ 0.05.

### 4.5. Consent

The study was approved by the Université Laval and Centre hospitalier universitaire (CHU) de Québec ethics committees and was performed in accordance with the principles of the Declaration of Helsinki. All participants provided written and informed consent. This study is derived from a registered clinical trial (NCT01343342).

## 5. Conclusions

Results of the present study demonstrate that the response of plasma TG to an ω-3 FA supplementation may be modulated through the effect of DNA methylation on expression levels of genes previously identified in a GWAS by our group (*IQCJ*, *NXPH1*, *PHF17* and *MYB*). Further research is needed to provide a better understanding of the relationship between these genes and TG metabolism.

## Figures and Tables

**Table 1:** Characteristics of the study sample before and after supplementation.

Characteristics	Before Supplementation	After Supplementation	*p* Value ^a^
Study population, n	210	208	-
Age, years	30.8 ± 8.7	-	-
Weight, kg ^b^	81.3 ± 13.9	81.6 ± 14.2	0.0009
BMI, kg/m^2^ ^b^	27.8 ± 3.7	27.9 ± 3.8	0.005
TG, mmol/L ^b^	1.21 ± 0.63	1.02 ± 0.52	<0.0001

Values are means ± standard deviation (SD) unless otherwise indicated; *p* Values were obtained using the Student’s *t*-test (TTEST) procedure (SAS Genetics v9.2); BMI = body mass index; TG = triglycerides; ^a^ *p* < 0.05 was considered significant; ^b^ *p* Values are for log10-transformed values.

**Table 2:** Selected polymorphisms in candidate genes from the genome-wide association study (GWAS) of the Fatty Acid Sensor (FAS) study (*n* = 210 individuals).

Gene	dbSNP No.	Sequence	Location	Genotype Frequency		
*IQCJ*	rs12497650	TTT[C/T]ATTG	Intron	C/C (*n* = 95) 0.4524	C/T (*n* = 96) 0.4571	T/T (*n* = 19) 0.0905
	rs4501157	ACA[G/T]TAA	Intron	G/G (*n* = 29) 0.1388	G/T (*n* = 89) 0.4258	T/T (*n* = 91) 0.4354
	rs13091349	TCT[C/T]CTC	Intron	C/C (*n* = 147) 0.7000	C/T (*n* = 56) 0.2667	T/T (*n* = 7) 0.0333
	rs2044704	TTT[C/G]TAG	Intron	C/C (*n* = 20) 0.0952	C/G (*n* = 68) 0.3238	G/G (*n* = 122) 0.5810
	rs1962071	AGC[A/C]GCC	Intron	A/A (*n* = 116) 0.5524	A/C (*n* = 74) 0.3524	C/C (*n* = 20) 0.0952
	rs7634829	TGT[A/G]TAA	Intron	A/A (*n* = 68) 0.3238	A/G (*n* = 101) 0.4810	G/G (*n* = 41) 0.1952
	rs2621294	TGC[A/G]AAG	Intron	A/A (*n* = 85) 0.4067	A/G (*n* = 90) 0.4306	G/G (*n* = 34) 0.1627
	rs6800211	AGG[C/T]GTC	Intron	C/C (*n* = 104) 0.4952	C/T (*n* = 90) 0.4286	T/T (*n* = 16) 0.0762
	rs17782879	TCC[A/G]TAT	Intron	A/A (*n* = 20) 0.0952	A/G (*n* = 88) 0.4190	G/G (*n* = 102) 0.4857
	rs1868414	CTG[C/T]GCC	Intron	C/C (*n* = 100) 0.4785	C/T (*n* = 81) 0.3876	T/T (*n* = 28) 0.1340
	rs2595260	AGG[C/T]ATC	Intron	C/C (*n* = 125) 0.5952	C/T (*n* = 66) 0.3143	T/T (n = 19) 0.0905
	rs6763890	ATG[A/T]CTT	Intron	A/A (*n* = 28) 0.1340	A/T (*n* = 85) 0.4067	T/T (*n* = 96) 0.4593
	rs9827242	TCA[C/T]AGT	Intron	C/C (*n* = 5) 0.0238	C/T (*n* = 68) 0.3238	T/T (*n*=137) 0.6524
	rs1449009 ^a^	CAA[C/T]ATT	Intron	A/A (*n* = 110) 0.5238	A/G (*n* = 77) 0.3667	G/G (*n* = 23) 0.1095
	rs2621309 ^a^	TTT[C/G]CTT	Intron	C/C (*n* = 114) 0.5429	C/G (*n* = 72) 0.3429	G/G (*n* = 21) 0.1000
	rs61332355 ^a^	AGG[A/C]AAT	Intron	A/A (*n* = 6) 0.0286	A/C (*n* = 64) 0.3048	C/C (*n* = 140) 0.6667
*NXPH1*	rs6956210	TTC[C/T]TTT	Intron	C/C (*n* = 14) 0.0667	C/T (*n* = 71) 0.3381	T/T (*n* = 125) 0.5952
	rs2107779	ATG[C/T]TGA	Intron	C/C (*n* = 69) 0.3286	C/T (*n* = 94) 0.4476	T/T (*n* = 47) 0.2238
	rs10273195	CTG[A/T]GGC	Intron	A/A (*n* = 134) 0.6381	A/T (*n* = 68) 0.3238	T/T (*n* = 8) 0.0381
	rs12216689	TGA[A/C]TGA	Intron	A/A (*n* = 108) 0.5143	A/C (*n* = 85) 0.T/T8	C/C (*n* = 17) 0.0810
	rs6963644	TGC[A/G]TTT	Intron	A/A (*n* = 0) 0	A/G (*n* = 32) 0.1524	G/G (*n* = 178) 0.8476
	rs17150341	AGG[C/T]ATT	Intron	C/C (*n* = 102) 0.4857	C/T (*n* = 88) 0.4190	T/T (*n* = 20) 0.0952
	rs1013868	TTC[A/G]CTG	Intron	C/C (*n* = 93) 0.4429	C/T (*n* = 95) 0.4524	T/T (*n* = 22) 0.1048
	rs12537067	CTA[A/G]CTC	Intron	A/A (*n* = 1) 0.0048	A/G (*n* = 33) 0.1571	G/G (*n* = 176) 0.8381
	rs4318981	CAT[C/T]ATA	Intron	C/C (*n* = 31) 0.1476	C/T (*n* = 88) 0.4190	T/T (*n* = 91) 0.4333
	rs17153997	GTG[C/T]GTA	Intron	C/C (*n* = 73) 0.3493	C/T (*n* = 92) 0.4402	T/T (*n* = 44) 0.2105
	rs7801099	AAC[A/G]ACA	Intron	A/A (*n* = 64) 0.G/T8	A/G (*n* = 105) 0.5000	G/G (*n* = 41) 0.1952
	rs4725120	ATA[A/G]AAG	Intron	A/A (*n* = 47) 0.2238	A/G (*n* = 99) 0.4714	G/G (*n* = 64) 0.3048
	rs1859275	GTG[A/G]CTA	Intron	A/A (*n* = 30) 0.1435	A/G (*n* = 87) 0.4163	G/G (*n* = 92) 0.4402
	rs10238726	CTC[A/G]TTC	Intron	A/A (*n* = 102) 0.4857	A/G (*n* = 84) 0.4000	G/G (*n* = 24) 0.1143
	rs1012960	GTT[A/T]CTT	Intron	A/A (*n* = 46) 0.2190	A/T (*n* = 118) 0.5619	T/T (*n* = 46) 0.2190
	rs11767429	ACA[A/G]GAG	Intron	A/A (*n* = 106) 0.5048	A/G (*n* = 82) 0.3905	G/G (*n* = 22) 0.A/T8
	rs4333500	AAG[G/T]TGG	Intron	G/G (*n* = 74) 0.3524	G/T (*n* = 105) 0.5000	T/T (*n* = 31) 0.1476
	rs7793115	GGA[A/G]GAG	Intron	A/A (*n* = 2) 0.0096	A/G (*n* = 37) 0.1770	G/G (*n* = 170) 0.8134
	rs7799856	CAA[A/C]AAA	Intron	A/A (*n* = 76) 0.3619	A/C (*n* = 88) 0.4190	C/C (*n* = 46) 0.2190
	rs7806226	ATA[A/C]TAT	Intron	A/A (*n* = 154) 0.7333	A/C (*n* = 46) 0.2190	C/C (*n* = 10) 0.0476
	rs13221144	CCT[C/T]ACG	Intron	C/C (*n* = 11) 0.0524	C/T (*n* = 75) 0.3571	T/T (*n* = 124) 0.5905
	rs17406479	TTG[G/T]TTT	Intron	G/G (*n* = 136) 0.6476	G/T (*n* = 69) 0.3286	T/T (*n* = 5) 0.0238
	rs10486228	AAA[C/T]TGA	Intron	C/C (*n* = 6) 0.0286	C/T (*n* = 64) 0.3048	T/T (*n* = 140) 0.6667
	rs17154569	TCT[A/G]AGA	Intron	A/A (*n* = 140) 0.6699	A/G (*n* = 64) 0.3062	G/G (*n* = 5) 0.0239
	rs4141002	CAC[C/T]TGT	Intron	C/C (*n* = 161) 0.7667	C/T (*n* = 46) 0.2190	T/T (*n* = 3) 0.0143
	rs7805772	GAG[A/G]TCC	Intron	A/A (*n* = 141) 0.6746	A/G (*n* = 56) 0.2679	G/G (*n* = 12) 0.0574
	rs2349780	ACA[A/G]TGG	Intron	A/A (*n* = 76) 0.3619	A/G (*n* = 108) 0.5143	G/G (*n* = 26) 0.1238
	rs2107474	CTT[C/G]AAC	Intron	C/C (*n *= 71) 0.3381	C/G (*n *= 100) 0.4762	G/G (*n *= 39) 0.1857
	rs11769942	TTC[C/T]GAC	Intron	C/C (*n *= 84) 0.4000	C/T (*n *= 95) 0.4524	T/T (*n *= 31) 0.1476
	rs6952383	CTA[A/T]TCT	Intron	A/A (*n *= 171) 0.8143	A/T (*n *= 36) 0.1714	T/T (*n *= 3) 0.0143
	rs6974252	GTA[A/G]TTG	Intron	A/A (*n *= 4) 0.0190	A/G (*n *= 51) 0.2429	G/G (*n *= 155) 0.7381
	rs10265408	CTG[C/G]ATG	Intron	C/C (*n *= 110) 0.5263	C/G (*n *= 83) 0.3971	G/G (*n *= 16) 0.0766
	rs2189904	TAC[C/T]TTT	Intron	C/C (*n *= 93) 0.4429	C/T (*n *= 95) 0.4524	T/T (*n *= 22) 0.1048
	rs2057862	AGA[C/G]TGT	Intron	C/C (*n *= 38) 0.1818	C/G (*n *= 95) 0.4545	G/G (*n *= 76) 0.3636
*PHF17*	rs2217023	GCA[C/G]ATG	nearGene-5′	C/C (*n *= 10) 0.0478	C/G (*n *= 61) 0.2919	G/G (*n *= 138) 0.6603
	rs4975270	TAC[A/G]CAT	Intron	A/A (*n *= 76) 0.3619	A/G (*n *= 88) 0.4190	G/G (*n *= 46) 0.2190
	rs11722830	CTT[A/G]TGG	Intron	A/A (*n *= 8) 0.0381	A/G (*n *= 73) 0.3476	G/G (*n *= 129) 0.6143
	rs12505447	CCT[C/T]GGG	Intron	C/C (*n *= 138) 0.6571	C/T (*n *= 65) 0.3095	T/T (*n *= 7) 0.0333
	rs6534704	GTA[A/T]CCT	Intron	A/A (*n *= 3) 0.0143	A/T (*n *= 26) 0.1238	T/T (*n *= 181) 0.8619
	rs13148510	CAA[C/G]AGC	3′ UTR	C/C (*n *= 193) 0.9190	C/G (*n *= 17) 0.0810	G/G (*n *= 0) 0
	rs13143771	ATA[C/T]AGT	nearGene-5′	C/C (*n *= 19) 0.0905	C/T (*n *= 81) 0.3857	T/T (*n *= 110) 0.5238
	rs13142964	TAA[C/G]AAC	nearGene-5′	C/C (*n *= 179) 0.8524	C/G (*n *= 31) 0.1476	G/G (*n *= 0) 0
*MYB*	rs9321493	GTA[C/T]ACT	Intron	C/C (*n *= 68) 0.3238	C/T (*n *= 97) 0.4619	T/T (*n *= 45) 0.2143
	rs11154794	AGC[C/T]TCC	Intron	C/C (*n *= 3) 0.0143	C/T (*n *= 47) 0.2238	T/T (*n *= 160) 0.7619
	rs210798	GAA[G/T]TCC	Intron	G/G (*n *= 75) 0.3571	G/T (*n *= 95) 0.4524	T/T (*n *= 40) 0.1905
	rs210936	CTT[C/T]TCC	nearGene-3′	A/A (*n *= 60) 0.2857	A/G (*n *= 98) 0.4667	G/G (*n *= 52) 0.2476
	rs7757388	ATA[A/G]AAG	Intron	A/A (*n *= 148) 0.7081	A/G (*n *= 55) 0.2632	G/G (*n *= 6) 0.0287
	rs210962	AGA[C/T]CCT	Intron	C/C (*n *= 125) 0.5981	C/T (*n *= 68) 0.3254	T/T (*n *= 16) 0.0766
	rs17639758	GTA[A/G]CAT	Intron	A/A (*n *= 0) 0	A/G (*n *= 11) 0.0524	G/G (*n *= 199) 0.9476
	rs1013891	TAC[A/G]GCA	Intron	A/A (*n *= 26) 0.1238	A/G (*n *= 94) 0.4476	G/G (*n *= 90) 0.4286
	rs2179308	GGT[A/G]TTG	Intron	A/A (*n *= 54) 0.2571	A/G (*n *= 106) 0.5048	G/G (*n *= 50) 0.2381

Genotype frequency was obtained using the ALLELE procedure (SAS Genetics v9.3); dbSNP = Single- Nucleotide Polymorphism (SNP) Database; ^a^ GWAS hit identified in the FAS study.

**Table 3:** Effects of genotype on the expression of transcripts associated with the *IQCJ*, *NXPH1*, *PHF17* and *MYB* genes (*n* = 30 individuals).

Gene	SNP	Transcript	*p* Value
*IQCJ*	rs17782879	NM_001042706.1	0.06
*NXPH1*	rs10486228	NM_152745.2	0.01
	rs11769942	NM_152745.2	0.07
*MYB*	rs11154794 *	NM_005375.2	0.09
	rs17639758	NM_005375.2	0.02

The General Linear Model (GLM) procedure (SAS v9.2) adjusted for age, sex and body mass index was used to test for the effects of genotype on the expression of transcripts; SNP = Single- Nucleotide Polymorphism; * Heterozygotes merged with rare homozygotes (dominant model).

**Table 4:** Significant associations of tagged single-nucleotide polymorphisms (SNPs) from genome-wide association study (GWAS)-associated genes with pre-supplementation methylation levels (*n* = 35 individuals).

Gene	SNP	CpG Site ^a^	Position	*p* Value ^b^
*IQCJ*	rs4501157	cg09784347	CHR 3, 158786963	0.0457
		cg17255703	CHR 3, 158787141	0.0077
		cg10461878	CHR 3, 158904717	0.0035
	rs2044704 ^c^	cg16975599	CHR 3, 158962761	0.0001 *
	rs1962071 ^c^	cg16975599	CHR 3, 158962761	0.0004 *
	rs7634829	cg17255703	CHR 3, 158787141	0.0025
	rs2621294	cg26659665	CHR 3, 158786242	0.0238
		cg10461878	CHR 3, 158904717	0.0301
		cg12919294	CHR 3, 158962196	0.0152
	rs17782879 ^c^	cg16975599	CHR 3, 158962761	0.0362
	rs1868414 ^c^	cg16975599	CHR 3, 158962761	0.0086
	rs2595260 ^c^	cg16975599	CHR 3, 158962761	0.0001 *
	rs9827242 ^c,d^	cg23982461	CHR 3, 158955027	0.0077
		cg16975599	CHR 3, 158962761	0.0056
	rs1449009 ^c,e^	cg16975599	CHR 3, 158962761	0.0001 *
	rs2621309 ^c,e^	cg16975599	CHR 3, 158962761	0.0001 *
	rs61332355 ^c,d,e^	cg16975599	CHR 3, 158962761	0.0002 *
*NXPH1*	rs6956210	cg06328127	CHR 7, 8469546	0.0089
		cg11642377	CHR 7, 8480779	0.0048
	rs2107779 ^c^	cg20378002	CHR 7, 8477029	0.0109
		cg08897422	CHR 7, 8666902	0.0059
		cg21001050	CHR 7, 8791686	0.0006
	rs12216689 ^c^	cg00852549	CHR 7, 8473457	0.0120
		cg17278735	CHR 7, 8476834	0.0127
		cg01933308	CHR 7, 8483569	0.0388
		cg13611822	CHR 7, 8483710	0.0404
	rs6963644	cg07462540	CHR 7, 8473279	0.0375
		cg00852549	CHR 7, 8473457	0.0327
		cg06444755	CHR 7, 8473990	0.0266
		cg20378002	CHR 7, 8477029	0.0123
		cg23075337	CHR 7, 8481460	0.0051
		cg22287800	CHR 7, 8634300	0.0245
		cg08897422	CHR 7, 8666902	0.0259
	rs1013868 ^c^	cg24785946	CHR 7, 8473234	0.0266
		cg00852549	CHR 7, 8473457	0.0103
		cg09225457	CHR 7, 8481460	0.0495
	rs4318981	cg06981279	CHR 7, 8477156	0.0452
		cg07511564	CHR 7, 8481036	0.0211
		cg01933308	CHR 7, 8483569	0.0474
		cg02614372	CHR 7, 8486611	0.0261
	rs7801099	cg24785946	CHR 7, 8473234	0.0238
		cg00852549	CHR 7, 8473457	0.0095
		cg09225457	CHR 7, 8479893	0.0289
	rs4725120	cg06981279	CHR 7, 8477156	0.0425
		cg01994275	CHR 7, 8480586	0.0122
	rs1859275	cg22339356	CHR 7, 8473385	0.0153
		cg01354961	CHR 7, 8473462	0.0148
		cg07511564	CHR 7, 8481036	0.0247
	rs1012960	cg07462540	CHR 7, 8473279	0.0437
		cg00852549	CHR 7, 8473457	0.0282
		cg06981279	CHR 7, 8477156	0.0093
	rs11767429 ^c^	cg00288806	CHR 7, 8476521	0.0287
		cg21001050	CHR 7, 8791686	0.0252
	rs4333500	cg00852549	CHR 7, 8473457	0.0479
		cg12597389	CHR 7, 8482235	0.0391
	rs7799856	cg24785946	CHR 7, 8473234	0.0343
		cg00399951	CHR 7, 8476098	0.0378
		cg00288806	CHR 7, 8476521	0.0270
		cg21001050	CHR 7, 8791686	0.0470
	rs13221144	cg08897422	CHR 7, 8666902	0.0448
	rs7805772 ^c^	cg12597389	CHR 7, 8482235	0.0445
	rs2349780 ^c^	cg19469357	CHR 7, 8474830	0.0264
		cg06056929	CHR 7, 8476128	0.0017
		cg00288806	CHR 7, 8476521	0.0167
		cg15285250	CHR 7, 8482107	0.0229
	rs11769942	cg09225457	CHR 7, 8479893	0.0113
	rs2057862	cg02124383	CHR 7, 8475198	0.0396
		cg20378002	CHR 7, 8477029	0.0017
		cg08897422	CHR 7, 8666902	0.0247
		cg21001050	CHR 7, 8791686	0.0048
	rs10265408	cg06328127	CHR 7, 8469546	0.0327
	rs10273195 ^d^	cg06981279	CHR 7, 8477156	0.0287
		cg06335867	CHR 7, 8482325	0.0467
		cg21806015	CHR 7, 8483249	0.0243
	rs12537067 ^d^	cg01994275	CHR 7, 8480586	0.0466
		cg06328127	CHR 7, 8469546	0.0346
	rs7793115 ^d^	cg22339356	CHR 7, 8473385	0.0435
	rs17406479 ^d^	cg26170604	CHR 7, 8482614	0.0143
	rs10486228 ^d^	cg20378002	CHR 7, 8477029	0.0435
		cg21001050	CHR 7, 8791686	0.0098
	rs17154569 ^d^	cg13611822	CHR 7, 8483710	0.0447
		cg06328127	CHR 7, 8469546	0.0298
	rs6952383 ^d^	cg00852549	CHR 7, 8473457	0.0464
		cg20378002	CHR 7, 8477029	0.0007
		cg23075337	CHR 7, 8481460	0.0102
		cg10443049	CHR 7, 8481537	0.0337
		cg12597389	CHR 7, 8482235	0.0331
		cg01933308	CHR 7, 8483569	0.0137
*PHF17*	rs4975270	cg19120496	CHR 4, 129731335	0.0493
		cg09164670	CHR 4, 129731835	0.0106
		cg26347359	CHR 4, 129759716	0.0069
	rs13148510	cg26766900	CHR 4, 129730719	0.0351
		cg11264547	CHR 4, 129731973	0.0399
	rs13143771	cg19120496	CHR 4, 129731335	0.0100
		cg26347359	CHR 4, 129759716	0.0095
	rs13142964	cg19120496	CHR 4, 129731335	0.0128
	rs11722830 ^d^	cg14617908	CHR 4, 129730872	0.0139
		cg09164670	CHR 4, 129731835	0.0133
		cg22868951	CHR 4, 129767588	0.0468
	rs12505447 ^d^	cg09164670	CHR 4, 129731835	0.0343
		cg09568483	CHR 4, 129753010	0.0337
	rs6534704 ^d^	cg17503252	CHR 4, 129733028	0.0303
		cg26347359	CHR 4, 129759716	0.0472
*MYB*	rs210936	cg07363239	CHR 6, 135502390	0.0016
		cg11579069	CHR 6, 135502794	0.0187
		cg01369646	CHR 6, 135502875	0.0091
	rs17639758	cg18253802	CHR 6, 135501780	0.0411
	rs1013891	cg00366934	CHR 6, 135503837	0.0471
		cg10574148	CHR 6, 135504383	0.0117
	rs2179308	cg00366934	CHR 6, 135503837	0.0441

The GLM procedure (SAS v9.2) adjusted for age, sex and BMI was used to test for the associations between tagged SNPs and methylation levels; ^a^ CpG site positions according to genome build 37; ^b^ *p* Values are derived from log10-transformed data; ^c^ Heterozygotes were merged with rare homozygotes (dominant model); ^d^ GWAS hit identified in the *FAS* study; ^e^ SNP showed genotype or gene-diet interaction effects on plasma triglyceride levels. * *p* Value remained significant after controlling for false discovery rate (MULTTEST procedure, SAS v9.2).

**Table 5:** Marginal and significant correlations between transcript expression and DNA methylation levels (*n* = 29 individuals).

Gene	Transcript	CpG Site	*p* Value
*IQCJ*	NM_001042706.1	cg034455716	0.0936
	NM_001042705.1	cg15736726	0.0230
		cg23982461	0.0141
*NXPH1*	NM_152745.2	cg0212438	0.0696
		cg06328127	0.0446
*PHF17*	NM_024900.3	cg26766900	0.0937
		cg04482257	0.0050
		cg00296291	0.0953
		cg12676803	0.0806
		cg26347359	0.0842
		cg27628849	0.0038
		cg17233452	0.0417
*MYB*	NM_005375.2	cg13400176	0.0567
		cg01369646	0.0240
		cg02127509	0.0496

The CORR procedure (SAS v9.2) adjusted for age, sex and BMI was used to test for the correlations between transcript expression and CpG site methylation levels.

**Table 6:** Marginal and significant correlations between pre-supplementation triglyceride levels and DNA methylation levels (*n* = 35 individuals).

Gene	CpG Site	*p* Value
*IQCJ*	cg09784347	0.0399
	cg034455716	0.0610
*NXPH1*	cg00852549	0.0459
	cg00399951	0.0404
	cg20378002	0.0368
*PHF17*	cg15389440	0.0070
	cg09863040	0.0317
	cg12832492	0.0050
	cg22922695	0.0686
	cg09884389	0.0629
	cg12204423	0.0943
	cg01223512	0.0424
*MYB*	cg18253802	0.0134
	cg07363239	0.0407

The CORR procedure (SAS v9.2) adjusted for age, sex and BMI was used to test for the correlations between transcript expression and CpG site methylation levels.
